# Optimal use of the AUDIT in screening and brief intervention programs: a decision theoretic approach (part of Economics of SBI symposium)

**DOI:** 10.1186/1940-0640-10-S2-O1

**Published:** 2015-09-24

**Authors:** Arnie Aldridge, Will Parish

**Affiliations:** 1 RTI International, RTP, USA

## Background

Current evidence suggests that screening and brief intervention (SBI) programs can be effective at reducing risky alcohol consumption. While validity and reliability have been demonstrated for existing screening instruments, little is known about how the diagnostic properties of an instrument influence the cost-effectiveness of SBI. We develop a decision theoretic framework to model this issue, and to measure the potential improvement in cost-effectiveness that could result from changes in screening instrument properties and other factors associated with SBI.

## Material and methods

To make use of our decision theoretic model, we obtain estimates of the input parameters by conducting a comprehensive review of the literature. In particular, we review the literature on the sensitivity and specificity of the Alcohol Use Disorders Identification Test (AUDIT), and then synthesize this evidence using meta-analytic methods. We also compile evidence on the cost of SBI, the QALY gains of SBI, and the prevalence of risky drinking among primary care patients. We assess uncertainty via Monte Carlo analysis. Finally, we conduct expected value of perfect information (EVPI) analyses to investigate the sources of decision uncertainty.

## Results

When QALYs are valued at a conservative $1,000/QALY our decision theory indicates an optimal AUDIT threshold score of 5. Our model indicates that employing the recommended threshold score of 8 results in about $4.50 per patient in foregone benefits. This suggests large aggregate foregone benefits with even modest sized patient populations. Gender-specific results are qualitatively similar, but reveal that the optimal SBI program is dramatically different across genders.

## Conclusions

Despite the relatively sound psychometric properties of the AUDIT, small differences in the sensitivity and specificity can have large impacts on the cost-effectiveness of SBI. Differences in the AUDIT's performance across males and females, as well as differences in the prevalence of risky drinking across genders, contribute heavily to the cost-effectiveness of SBI as well.

